# Associations between impulsivity, aggression, and suicide in Chinese college students

**DOI:** 10.1186/1471-2458-14-551

**Published:** 2014-06-03

**Authors:** Lin Wang, Chang Zhi He, Yun Miao Yu, Xiao Hui Qiu, Xiu Xian Yang, Zheng Xue Qiao, Hong Sui, Xiong Zhao Zhu, Yan Jie Yang

**Affiliations:** 1 Psychology Department, Public Health Institute, Harbin Medical University, 157 Baojian Road, Nangang, Harbin 150081, China; 2 Medical Psychological Institute, Second Xiangya Hospital, Central South University, Changsha, Hunan, China

**Keywords:** Suicidal ideation, Suicidal attempters, Impulsivity, Aggression, University students, Correlations

## Abstract

**Background:**

Although there are accumulating data regarding the epidemiology of suicide in China, there are meager data on suicidal ideation and attempts among college students. Interestingly, elevated impulsivity is thought to facilitate the transition from suicidal thoughts to suicidal behavior. Therefore, the objective of this research was to identify the associations between suicide and the personality factors of impulsivity and aggression.

**Methods:**

This study’s sampling method employed stratified random cluster sampling. A multi-stage stratified sampling procedure was used to select participants (n = 5,245). We conducted structured interviews regarding a range of socio-demographic characteristics and suicidal morbidity. The Patient Health Questionnaire depression module (PHQ-9) was used to acquire the information about thoughts of being better off dead or hurting themselves in some ways during the past two weeks. The impulsivity symptoms in this study were assessed with the BIS-11-CH (i.e., the Chinese version of the BIS-11), and the Aggressive symptoms were assessed with the BAQ. The statistical package for social science (SPSS) v.13.0 program (SPSS Inc., Chicago, IL, USA) was used for statistical analysis. Socio-demographic variables such as ethnic and gender were compared between groups, through the use of *χ*^2^ tests. The nonparametric test (k Independent Sample test, Kruskal-Wallis H) was performed to determine differences between the personality factors of impulsivity and aggression and suicide.

**Results:**

In total, 9.1% (n = 479) of the 5,245 students reported they have ever thought about committing suicide; and 1% (n = 51) reported a history of attempted suicide (attempters). The analyses detected significant differences in scores on cognitive impulsivity (p < 0.01), when comparing individuals who only had suicidal ideation and individuals who had attempted suicide. Moreover, significant differences were found between ideators only and attempters on scores of self-oriented attack (p < .001).

**Conclusions:**

Suicidal ideation is prevalent among Chinese university students. Students with high aggression scores were more susceptible to committing suicide. Scores on self-oriented attack and cognitive impulsivity may be important factors for differentially predicting suicide ideation and suicide attempts.

## Background

Suicide is the leading cause of deaths among all the deaths due to injuries. The World Health Organization has acknowledged that suicide behavior is a major global public health problem in every country, with approximately one million people dying from suicide each year [1-3]. In China, which has almost 1.3 billion inhabitants, suicide is among the top five causes of death. Suicide attempts are even more common, and for every completed suicide there are 8–25 times as many suicide attempts; nearly 2 million people attempt suicide each year in our nation [4]. Previous studies have reported that the prevalence of suicidal ideation and suicide attempts in university students in China is nearly 21.5% [5].

Suicidal behavior is often seen as a continuum extending from wishing to be dead or passive ideation, through thoughts and plans about harming oneself, to suicide attempts and completed suicide [6]. The significance of suicide risk varies from low severity (thoughts of death) to the highest severity (suicidal ideation with a plan or recent suicidal attempt) [7]. Suicidal ideation means thinking about suicide without actually making plans to commit suicide [8]. Studies have found that more than 80% of suicide deaths had exhibited various forms of suicidal thoughts before their action. It has been suggested that it is necessary to obtain more precise knowledge about suicide related predictors to identify persons at increased risk for suicide [9].

It is reported that compared with the general public, individuals with high impulsivity will more easily act on their suicidal thoughts and make a suicide attempt. Elevated impulsivity is thought to facilitate the transition from suicidal thoughts to suicidal behavior [10,11]. Indeed, we have learned from previous studies that there are strong links between impulsivity and suicidal behavior [12-14]. Other studies have suggested that future research should continue to distinguish impulsivity-related traits that predict suicide, and that models of suicide risk should be revised accordingly [10,15,16].

In addition to impulsiveness, aggressiveness is a trait that is associated with suicidal behavior. Several studies have reported a relationship between aggression and suicide, such as Mathias [17], who found that that girls with multiple suicide attempts acted more impulsively, and had higher self-ratings of depression and aggression than girls with either one or no suicide attempts [17]. Interestingly, a cross-sectional survey did not find aggression to be a predictor of attempted suicide. Aggression seems to have only a minor role in suicidal behavior in schizophrenia [18]. Aggression is complex, and it is unclear whether increased aggression correlates with suicide.

University students are a particular group of people who are in a critical transitional period. Many college students experience frustration because of the pressure of competition for good grades and the failure to find work [19]. As a result, depression is common in university students, and in extreme cases, it may lead to suicide. The risk for suicide is often higher among college students, compared with same-age noncollegiate peers, and it may be exacerbated by quality of social support and interaction [20]. However, little is known about the prevalence of and factors associated with suicide ideation and suicide attempts among college students in China.

To our knowledge, there is no study that has directly tested the associations between impulsivity and aggression, and it is not certain if the personality traits of impulsivity and aggression increase the risk of suicide attempts among persons considering suicide. Currently, there are only two studies have examined the relationship between the impulsivity and suicide. Brezo found comparable levels of impulsivity among suicide attempters and suicide ideators [21]. Klonsky et al., who hypothesized that elevated impulsivity distinguishes suicide attempters from ideators, were unable to support this hypothesis using a multidimensional measure of impulsivity [10]. The current strand of research would, ideally, determine if impulsivity and aggression can prospectively predict suicidal behavior among a sample of Chinese college students who are suicide ideators. The researchers use a cross-sectional design to compare impulsivity and aggression in suicide attempters and ideators.

In the present study, we evaluated the hypothesis that elevated impulsivity and aggression may distinguish between individuals who have attempted suicide from those who have considered suicide but never attempted it. It seems that impulsivity and aggression can lead to suicide, but it is unclear whether increased impulsivity and aggression are correlated with suicidal attempts. Thus, it is important that studies that can accurately measure impulsivity and aggression are conducted in China with large sample sizes. Therefore, the current study was designed to explore more effectively the association between impulsivity, aggression, and suicide in a study with a sufficient sample from which to draw conclusions.

## Methods

### Study population

The study was conducted in Harbin, the capital of Heilongjiang Province in northeastern China. Harbin is ranked as the 10th largest city in China, and it serves as a key political, economic center in northeastern China.

There are two main types of universities in China: one is the key national universities, and the other is general institutions. There are 14 universities in Harbin; three of them are key national universities and 11 are general institutions.

### Sampling and sample

This study employed stratified random cluster sampling to acquire a representative sample of university students in China from two key national universities and four general institutions, which was randomly selected. The distribution of the samples from each university was calculated as the proportion of students who attended the two main types of universities. The sampling frame consisted of university students in full-time studies during the 2007–2008 academic year. The sampling frame was stratified into five grades (i.e., first, second, third, and fourth years, and postgraduate year), and classes were randomly selected from each grade.

In this way, 6,000 students were selected from a total of 274,041 students in Harbin. This study was conducted using an exploratory design with quantitative data collected at the universities. Study participants who had an experience of suicidal ideation were categorized as belonging to the suicide ideator group (479 students, 289 were female, 190 were male); these individuals were invited to complete the Barratt Impulsivity Scale (BIS) and an aggression questionnaire. All participants were free of any organic brain lesions and of any type of personality disorder; none currently had a psychiatric disorder.

### Procedure

Approval for this study was granted by the Ethics Committee of Harbin Medical University, the Education Committee of Heilongjiang Province, and the institutional review committees of each of the selected universities. The selected students were told about the purposes, benefits, and possible adverse effects of the study, and they were then invited to participate. Written informed consent for participation in the study was obtained from the participants.

All participants had been free of alcohol or drugs for at least 72 h prior to the test. Participants were advised that the questionnaires should be finished within 15 min. The investigation was timed so that the testing was not done at the beginning or the end of the semester.

### Measures

The demographic variables included gender, age (16–43 years), ethnicity, student major, year of study, satisfaction with major, relationship with parents, educational levels of parents, university classification, and family economic situation. We defined < 800 ¥ as low income (poor), 800–2000 ¥ as middle income (moderate), and >2000 ¥ as high income (good), which are the criteria used by the State Labor and Social Security Department.

Depressive symptoms in this study were assessed with the 21-item BDI [22]. The BDI, created by Dr. Aaron T. Beck, is a self-report inventory, and one of the most widely used instruments for measuring the severity of depression. It can be used as a depression screening in non-clinical people. The BDI is a reliable and valid instrument [23]. Each statement in this inventory has a possible score range of 0 to 3, with the total score being 63. A score of 0 to 4 is considered as normal, 5 to 13 border line clinical depression, 14 to 20 moderate depression, and 21 to 63 severe depression. The internal consistency (Cronbach α) was high in many countries, ranging from 0.75 to 0.88 [24]; in this study the Cronbach α is 0.851. The cut-off score for depression in this study was 14, as has been chosen in -several previous studies [24,25].

We determined suicidal ideation with a two-tier process. The first-tier screen consisted of the suicide item of the Patient Health Questionnaire depression scale (PHQ-9), which asks about thoughts of being better off dead or hurting oneself in some way during the previous 2 weeks [26]. Students who reported death or suicidal thoughts during several days or more were administered the suicide module of the mini interview. They were asked about general suicidal ideation with the question, “Have you ever thought about committing suicide?” Each student also was asked about serious suicide ideation, suicide plans, and suicide attempts, using items taken directly from the National Comorbidity Survey, with one minor addition to the item about suicide plans [27]. These items were: “Have you ever seriously thought about committing suicide?” “Have you ever made a plan for committing suicide, or even taken steps to prepare for this plan?” These questions have demonstrated substantial reliability. Students endorsing any question in this section were classified as suicide ideators. One item, “Have you ever attempted suicide?” was used to assess lifetime history of attempted suicide. Participants endorsing this item were considered to have a history of attempted suicide.

Impulsivity is a major personality and temperament dimension in several theoretical models [28]. The Chinese version of the Barratt Impulsiveness Scale-11 (i.e., the BIS-11-CH) was modified from the original 30-item BIS-11 by removing five items showing poor item-total correlations. It therefore consists of 25 self-report items, each of which is rated on a four-point Likert scale; the BIS-11-CH is purported to measure stable, long-standing, impulsive-behavior patterns.

The BIS-11-CH consists of three factors: “inability to plan (absence of weighing the long-term consequences of actions),” “cognitive impulsivity (rapid shifts in attention and impatience with complexity),” and “motor impulsivity (acting impetuously).” The BIS-11-CH’s internal consistency, 0.83 among the 25 items, was found to be satisfactory.

Aggression includes feelings of anger and hatred that may result in threatening or violent behavior. Aggressive symptoms in this study were assessed with the Buss–Perry Aggression Questionnaire [29], which consists of 30 items. The revised Chinese version includes five subscales: physical aggression, verbal aggression, anger, hostility, and self-oriented attack [30]. There is no consensus as to a precise definition of the concept of “aggression,” but this questionnaire is considered to allow comparisons to be made between participants and controls on internal aggressiveness. The internal consistency of this inventory is 0.85, which indicates that the BAQ can be used with the Chinese population.

All items of the BIS and the aggression questions are answered on a five-point Likert scale (i.e., Never, Rarely, Occasionally, Often, Almost always). Items were rated from 1 to 5, with 5 indicating the most impulsive and aggressive response.

### Data analysis

The Statistical Package for the Social Sciences (SPSS) v.13.0 was used to perform the statistical analyses (SPSS Inc., Chicago, IL, USA). All tests were two-tailed, and the significance level was set at p < 0.05. Sociodemographic variables, such as ethnicity and gender, were compared between groups by *χ*^2^ tests. Because there were three groups, nonparametric tests (the K Independent Sample test, and the Kruskal–Wallis test) were performed to determine group differences. Adjustments were made for demographic characteristics (i.e., age, gender, ethnicity, and study year) at baseline. Results are presented as median values, and non-skewed data are presented as mean ± standard deviation.

## Results

### Participants’ sociodemographic data

A total of 6,000 questionnaires were distributed, of which 5,479 questionnaires were returned (response rate, 91.3%). After excluding 234 invalid questionnaires (i.e., those in which >20% of questions were unanswered), 5,245 students had completed the screening questionnaires during the survey (completion rate, 87.4%). Of these, 2,563 (48.9%) were male and 2,682 (51.1%) were female. There were 4,878 (93.0%) students of the Han nationality, and 367 (7.0%) of other nationalities. The average age of the participants was 21.3 years (SD = 2.2), with a range of 16–43 years. Their families’ economic situations were as follows: 354 (6.8%) good, 3,542 (67.5%) moderate, and 1,349 (25.7%) poor. Their relationships with their parents were as follows: 4,229 (80.6%) good, 785 (14.9%) moderate, and 231 (4.5%) poor. Regarding the students’ majors, the distribution was as follows: 790 (15.1%) science; 662 (12.6%) management; and 419 (7.9%) other majors.

### Descriptive statistics

In total, 9.1% (n = 479) of 5,245 students reported they have ever thought about committing suicide, and nearly 1% (n = 51) reported a history of attempted suicide (attempters). Just over 90% of the sample (n = 4,743) denied a history of suicidal ideation and attempts. Table 1 shows the prevalence rates of suicide ideation for female and male students. The figures indicate that female rates are generally higher than the male rates across the suicide measures. The rates of suicidal thoughts and attempts are not similar to the rates reported by previous studies in the China. Other sociodemographic variables of the sample are presented in Table 1.

**Table 1:** Socio-demographic factors of the Chinese university students studied (n = 5,245)

**Variables**	**Totle**	**Not suicide (N = 4743)**	**Ideators (N = 439)**	**Attempts (N = 51)**	***P***
Gender					
Female	2682 (48.9%)	2261 (47.67)	262 (59.68)	35 (68.63)	< .0001
Male	2563 (51.1%)	2482 (53.33)	177 (40.32)	16 (31.37)	
Ethnic					
Han	4878 (93.0%))	4410 (92.98)	408 (92.94)	49 (96.08)	0.6878
Other	367 (7%)	338 (7.02)	31 (7.06)	2 (3.92)	
Family economic situation					
Good	354 (6.8%)	326 (6.87)	22 (5.01)	6 (11.76)	0.2021
Moderate	3542 (67.5%)	3213 (67.74)	292 (66.51)	31 (60.78)	
Poor	1349 (25.7%)	1204 (25.38)	125 (28.47)	14 (27.45)	
Parent relationships					
Good	4229 (80.6%)	3889 (81.99)	302 (68.79)	28 (54.90)	< .0001
Moderate	785 (14.9%)	667 (14.06)	102 (23.23)	15 (29.41)	
Average	123 (2.3%)	100 (2.11)	19 (4.33)	4 (7.84)	
Poor	231 (4.5%)	87 (1.83)	16 (3.64)	4 (7.84)	
Major					
Science	790 (15.0%)	718 (15.14)	60 (13.67)	10 (19.61)	0.0006
Engineering	2406 (45.9%)	2201 (46.41)	193 (43.96)	12 (23.53)	
Medicine	578 (11.0%)	504 (10.63)	67 (15.26)	7 (13.73)	
Literature	380 (7.2%)	330 (6.96)	40 (9.11)	10 (19.61)	
Management	662 (12.6%)	607 (12.80)	47 (10.71)	8 (15.69)	
Others	419 (7.9%)	383 (8.08)	32 (7.29)	4 (7.84)	
Satisfaction with major					
Yes	2104 (40.1%)	1950 (41.11)	141 (32.12)	13 (25.49)	< .0001
Moderate	2775 (53.0%)	2498 (52.67)	249 (56.72)	28 (54.90)	
No	354 (6.7%)	295 (6.22)	49 (11.16)	10 (19.61)	

### Variance analyses

No significant association was found between the three groups on ethnicity or family economic situation (see Table 1). Gender differences were found in the prevalence of suicidal ideation and suicide attempts. Female students were more likely than male students to be in the ideator-only group (59.68% of female students to 40.32% of male students) or the attempter group (68.63–31.37%). Other results were presented in Table 1.

Table 2 presents comparisons of the scores on the BIS-11-CH and BAQ among the three groups. Because of missing values, only 5,233 observations could be used in the analyses. To investigate the difference between the three groups on impulsivity and aggression, nonparametric tests were performed to determine which symptoms of impulsivity and aggression contributed most to the differences between the three groups.

**Table 2:** Comparisions of scores of BIS-11-CH and BAQ between the three groups

	**Not suicide**	**Ideators**	**Attempts**	***P***
	**Mean ± SD**	**Mean ± SD**	**Mean ± SD**	
Cognitive impulsivity	36^a^ ± 4.1	35^a^ ± 4.5	33^b^ ± 5.4	0.0032
Planning impulsivity	36^a^ ± 6.1	34^b^ ± 6.2	32^b^ ± 6.6	< .0001
Motor impulsivity	22^a^ ± 5.8	25^b^ ± 6.3	26^b^ ± 7.7	< .0001
Physical aggression	23^a^ ± 16.7	27^b^ ± 18.0	30^b^ ± 19.0	< .0001
Hostility	26^a^ ± 15.8	36^b^ ± 16.4	40^b^ ± 17.9	< .0001
Verbal aggression	29^a^ ± 15.7	35^b^ ± 17.5	36^b^ ± 16.2	< .0001
Self-oriented attack	20^a^ ± 16.2	33^b^ ± 19.9	43^c^ ± 20.6	< .0001
Anger	27^a^ ± 18.2	37^b^ ± 19.9	42^b^ ± 21.6	< .0001

Note. This table depicts results from the nonparametric test (k Independent Sample test, Kruskal-Wallis H). Median ± standard deviation for individual scores*, P < 0.05* considered singnificant. The superscript letter (a, b and c) means that there are significant differences between them (*P < 0.05*).

We compared scores on impulsivity and aggression between non-suicide, suicide ideators (ideators only), and suicide attempters (Table 2). The K Independent Sample test found significant main effects of groups and scales. There were significant differences between the group of attempters and the other two groups on impulsivity. Students who never thought of suicide scored significantly lower than ideators only and attempters on motor impulsivity (p < .05) and planning impulsivity (p < .05). Significant differences in cognitive impulsivity (p < .01) were found between ideators only and attempters. Suicide attempters were found to have higher scores on motor impulsivity and planning impulsivity when compared with the other groups. We also found that suicide attempters had lower scores on cognitive impulsivity and non-planning impulsivity than the other groups. There were significantly higher scores for suicide ideators on motor impulsivity, as well as higher scores for suicide attempters on motor impulsivity related to the personality subscales of the impulsivity.

With respect to aggression, students who were never suicidal scored significant lower than ideators only and attempters on all subscales of aggression. Those who were never suicidal scored significant lower than ideators only and attempters on physical aggression (p < .05), hostility (p < .05), verbal aggression (p < .05), and anger (p < .05). Moreover, significant differences were found between ideators only and attempters on scores of self-oriented attack (p < .001). However, significant differences were only found between the attempter and ideators-only groups for self-oriented attack. Furthermore, the suicide ideator and attempter groups had higher average scores for aggression expression.

## Discussion

The current study is one of a series of large epidemiological studies being undertaken regarding suicide among Chinese university students. Compared with the previous research, our study has made changes to the study design. These changes include the different region studied, different measurement tools, different methods, and different appraisal standards.

The study found that the prevalence rate of suicidal ideation in this demographic group was 9.1%. This rate does not align with the findings of previous studies, which reported that the prevalence rate of suicidal ideation and attempts in university students in China was nearly 21.5% [5,9]. This disparity may arise from the use of different regions, different sample sizes, different methods, and different appraisal standards. For a Chinese person, for example, attending a university is an important transitional life stage. Undergoing such a transition may lead to an increased risk of suicide behaviors. However, the prevalence rate of suicide ideation in the current study is a common incidence rate, similar to that in the general population, but lower than the rural population [31].

The analyses of sociodemographic factors were limited to gender, different majors, and different satisfaction with major. As expected for gender, we observed several sex differences in suicidality in our sample. Female students were more likely than male students to be in the ideator-only or attempter groups. Related studies have reported a similar pattern: that both the suicide attempt and suicidal ideation rates were higher in women [32,33]. However, Liu Guo Hua et al. reported different results in which 20.6% of male college students had a rate of suicide attempts and suicidal ideations that was slightly higher than female students (18.0%) [34]. However, that study only included students in the first grade of college, while our study included all grades of students. Moreover, we found that differences in majors and differences in satisfaction with majors were related to the prevalence of suicidal ideation and suicide attempts. Students who were satisfied with their major exhibited less suicidal ideation and behavior than those who were not satisfied. In China, a student’s major generally is determined by his/her parents. When students do not like their major, they have lower interest, passion, learning motivation, and academic performance. Thus, the students who are dissatisfied with their major might have depressive symptoms, which could result in suicide. The same results were obtained from a study by Bayram et al. [35].

We also found several differences in suicidality that were associated with students’ relationships with their parents. Students who had a poor relationship with their parents were more likely to have suicidal ideation, and even to commit suicide. Zhou Fang et al. showed that there is strong relationship between parental relationship and the suicidal ideation of Chinese college students, which is consistent with our results. The reason may be that a harmonious family and a harmonious parent–child relationship decrease the risk of suicidal ideation [36]. Zhou Fang’s study showed that a harmonious parent–child relationship has a great influence on the formation of the normal psychology of college students in China. In addition, there is research on China that found that poor parental relationships or bad behavior by parents in the family can directly lead young people to commit suicide [37]. Furthermore, several cross-sectional studies, employing retrospective self-reports, have suggested that parental relationships during childhood are associated with mental health outcomes in later life, including depression, anxiety, and self-harm [38,39].

The results of the study support our hypothesis that elevated impulsivity and aggression would distinguish individuals who have attempted suicide from those who have considered suicide but never attempted it. We examined the association of impulsivity and aggression with suicide ideation and suicide attempts in a large sample of university students in China. It has been reported that trait impulsivity could increase the chance that suicidal thoughts will lead to suicidal behavior, and generally speaking, that suicide is an expression of aggression. Therefore, it was hypothesized that high impulsivity and aggression would differentially predict individuals who have attempted suicide from those who only have suicidal ideation.

Our findings partly support this hypothesis, in that there were significant differences between the students who only had suicide ideation and students who had attempted suicide on cognitive impulsivity and self-oriented attack. Specifically, the BIS-11-CH and the BAQ questionnaires were used with a large sample to measure differences in impulsivity and aggression among students with histories of either suicide attempts or suicide ideation only and students who had never been suicidal. There was no significant difference between the suicide ideation-only individuals and suicide attempters on most subscales of impulsivity and aggression. Therefore, we propose that multidimensional models of impulsivity and aggression are necessary to explicate the links between impulsivity, aggression, and suicide. Because the BIS-11-CH and the BAQ scales empirically overlap with many aspects of impulsivity and aggression, we cannot conclude whether all or just certain aspects of impulsivity or aggression fail to distinguish attempters from ideators, based on these findings alone.

The BIS-11-CH measure of impulsivity gives us a better understanding of impulsivity in suicide. One dimension, cognitive impulsivity (rapid shifts in attention and impatience with complexity), differentiated between the ideators only and attempters. One might speculate that the typical person is less impulsive than people who commit suicide; however, the results of the current study suggest that cognitive impulsivity is higher in the non-suicide group. Furthermore, decreased cognitive impulsivity differentiated between individuals with histories of either suicidal thoughts or behavior from individuals who had never been suicidal. This indicates the people who could not shift rapidly in attention, and typically follow the prescribed order are more likely to commit suicide. Even when participants prepare sound plans for the future, they could become nervous and anxious if things did not develop as expected. Because such people tend not to be flexible and are very rigid in handling problems, they will be consistently puzzled by problems. Therefore, it is difficult for them to find ways to experience emotional catharsis. There are some similar reports of this relationship in previous studies. The relationship between impulsivity and suicide ideation found in research from abroad indicates that impulsivity is an independent risk factor for suicide [40]. Aldis et al., who investigated the correlates and predictors of suicidal behaviors among 900 young offenders in detention centers in South Australia, suggested that impulsiveness is a common underlying factor [41].

There have been only a few other published reports concerning the relationship between aggressive behavior and suicide in student samples. Aggression and suicide are related, but the nature of this relationship remains unclear, because the literature is confusing and contradictory. This is probably because of the difficulty in defining and separating out these concepts, and the fact that there is much overlap between them. Moreover, the number of participants in each of those studies was too small to detect the relationship between aggressive behavior and suicide.

Indeed, aggression has been consistently reported to be elevated among individuals with histories of either suicide attempts or suicide ideation. Thus, the results of the current study support the assertion that it is important to explore further the relationship between aggressive personality and the tendency to commit suicide. In addition, the mean levels of overall aggressiveness on the BAQ were significantly lower among participants who had never been suicidal. Higher levels of anger, hostility, and self-oriented attack may be attributable to the increased suicidal tendency among those individuals. This indicates that a person with a history of either suicide attempts or suicide ideation will tend to show higher verbal and physical aggression, anger, hostility, and self-oriented attack. It is widely suspected that suicidal individuals possess symptoms of motor retardation and of being low in morale. Therefore, in facing problems, they will exhibit weak reactions and poor concentration, and only rarely will they demonstrate verbal or physical aggression. However, only elevated self-oriented attack distinguished suicide attempters from suicide ideators, which suggests that elevated scores on self-oriented attack can differentiate persons with histories of suicidal thoughts from persons who had a suicide attempt. High self-oriented attack may increase the chances that individuals with suicidal ideation will act on their suicidal thoughts and make an attempt. Previous studies have shown that aggressive individuals tend to have high suicidal tendencies, and this trait explains why suicidal persons also tend to receive high scores *vis-à-vis* the self-oriented attack subscale. In fact, it is often the case that some people with histories of either suicide attempts or suicide ideation will suddenly become furious and indignant, but later feel disappointed and hate themselves for it; they may even feel rage about their sudden anger. This anger is symptomatic not only of a failure to solve problems, but also of feeling vulnerable. Any barrier preventing them from realizing goals will be thought as a personality defect and this, in turn, will usually stimulate their underlying sense of inferiority.

Impulsivity and aggression are two of a few psychological characteristics that play an important role in suicide assessments and determining level of risk [14]. Our findings can have practical applications for understanding suicide risk, and making clinical assessments for treatment. These variables not only have predictive value but should also be considered as appropriate targets for interventions to reduce suicide risk. To prioritize treatment targets and implement safety precautions, identifying patients at moderate and high risk for suicide is necessary [10]. Results of our study suggest that the diminished ability to make rapid shifts in attention, impatience with complexity, and an elevated tendency toward self-oriented attack are risks for suicidal thoughts that can lead to suicidal behaviors. As many psychiatric patients experience suicidal ideation, it is important to assess these aspects of impulsivity and aggression, which can play an important role in assessing suicide risk.

However, there are several limitations in our study. First, our study was carried out in one province with nearly 38 million people and many nationalities. Second, although we used a large non-clinical sample of college students, there are many people who were not included in our study. Consequently, future studies should examine whether the findings can be replicated in other samples, including samples drawn from clinical and geriatric populations.

## Conclusions

Suicidal ideation is prevalent among Chinese university students, and students with high aggression scores appear to be more susceptible to committing suicide. Moreover, significant differences were found between individuals who only experienced suicidal ideation and individuals who had attempted suicide in terms of their scores on self-oriented attack and cognitive impulsivity. The scores of self-oriented attack and cognitive impulsivity may be important factors for differentially predicting between individuals with suicide ideation and those who have made or will make suicide attempts.

## Competing interests

This research was supported by the overseas scholars program of the Education Department of Heilongjiang Province (1151hz043), the China Medical Board of New York Inc. (05–813), a Provincial Science Foundation Council general project grant (D200806), and the National Natural Science Foundation of China (31271093) to Prof. Yanjie Yang; and the project of science and technology of Heilongjiang provincial education department (12541496) to Lin Wang. We do not hold any stocks or shares in an organization that may in any way gain or lose financially from the publication of this manuscript, either now or in the future.

## Authors’ contributions

LW and CZH equally contributed to this work. They carried out the epidemiological investigation reported in the article, conceived of the study, and participated in its design and coordination, and drafted the manuscript. XHQ participated in the design of the study and performed the statistical analyses. XXY carried out the epidemiological investigation of the article, and participated in the design of the study. ZXQ participated in the design of the study. HS performed the statistical analyses. XZZ and YMY carried out the epidemiological investigation. YJY is the corresponding author of the article, and she conceived of the study, and participated in its design and coordination, and helped to draft the manuscript. All authors read and approved the final manuscript.

## Pre-publication history

The pre-publication history for this paper can be accessed here:

http://www.biomedcentral.com/1471-2458/14/551/prepub
